# Case Report: Favorable Response and Manageable Toxicity to the Combination of Camrelizumab, Oxaliplatin, and Oral S-1 in a Patient With Advanced Epstein–Barr Virus-Associated Gastric Cancer

**DOI:** 10.3389/fonc.2021.759652

**Published:** 2022-01-13

**Authors:** Wanrui Lv, Ke Cheng, Xiaofen Li, Lusi Feng, Hancong Li, Jia Li, Chen Chang, Dan Cao

**Affiliations:** Department of Medical Oncology, Cancer Center, West China Hospital, Sichuan University, Chengdu, China

**Keywords:** EBV-associated gastric cancer, camrelizumab, immunotherapy, programmed cell death ligand-1 (PD-L1) positive, favorable response, manageable toxicity

## Abstract

Some pertinent studies have demonstrated that Epstein–Barr virus-associated gastric cancer (EBVaGC) patients showed a favorable clinical outcome to immunotherapy and Epstein–Barr virus (EBV)-positive status might be a potential biomarker for immunotherapy in gastric cancer (GC). However, knowledge of given exposure to EBVaGC to the first-line immunotherapy is largely inadequate. Moreover, whether camrelizumab can be as effective as other PD-1 inhibitors in the treatment of advanced EBVaGC has not been reported. We report a case of advanced EBVaGC patient with a positive expression of PD-L1, enriched PD-L1+CD68+macrophages, and high TMB who had a long-term partial response and manageable toxicity to the combined approach of camrelizumab (a novel PD-1 inhibitor) and oxaliplatin plus oral S-1 (SOX). As the first-line treatment of advanced EBVaGC patients, camrelizumab combined with SOX regimen may provide a novel combined approach with favorable response and manageable safety. Combination of multiple biomarkers could have a higher effective predictive capacity to immunotherapy. Integrated treatment (chemo-immunotherapy and radiotherapy) might be the optimal strategy for patients with oligometastasis. It deserves prospective research to further validate the efficacy.

## Introduction

Gastric cancer (GC) remains a significant burden worldwide with an estimated 1,089,103 new cases and 768,793 deaths in 2020 (1). The prognosis and survival are much worse for advanced GC. Based on the CheckMate 649 trial (2), nivolumab combined with fluoropyrimidine and oxaliplatin has been adopted as the first-line treatment for advanced GC patients with HER2 overexpression negative and PD-L1 CPS ≥5 in the NCCN guideline (3). Epstein–Barr virus-associated gastric cancer (EBVaGC), as one of four subtypes of gastric carcinoma, accounts for ∼9% of GC (4, 5). Although very limited data in EBVaGCs are known, some pertinent studies have demonstrated that EBVaGC patients showed a favorable clinical outcome to immunotherapy and EBV-positive status might be a potential biomarker for immunotherapy in GC. Kim et al. showed a striking result that compared with the overall response rate (ORR) of 85.7% in microsatellite instability-high metastatic gastric cancer (mGC), ORR in EBV-positive mGC is 100% (6). In a prospective observational study, 66.7% of EBVaGC patients showed a partial response (PR) after combined immunotherapy (7). Plausible explanations contributing to favorable efficacy of the anti-PD-1 antibody in EBVaGC mainly involve the EBV-related cancer-intrinsic characteristics, including the tumor-associated immune cell-rich phenotype as well as the overexpression of PD-L1. Since late-stage EBVaGC patients receiving treatments only comprise ∼3% of GC cases, knowledge of given exposure to EBVaGC to the first-line immunotherapy is largely inadequate. Trials of applying nivolumab and pembrolizumab to the first-line treatment of advanced GC have been carried out one after another and achieved corresponding success (2, 8). Whether the aforementioned observations in these anti-PD-1 antibodies may analogously be extended to advanced GC treated with camrelizumab, especially EBVaGC, has not been reported. We herein report a case of a metastatic EBVaGC patient with an overexpression of PD-L1, enriched PD-L1+CD68+macrophages, and high TMB who had early tumor shrinkage, deep response, long-term duration of response, and manageable toxicity to camrelizumab, a novel PD-1 blockade, in combination with standard chemotherapy as a first-line setting.

## Case Presentation

**Figure 1:**
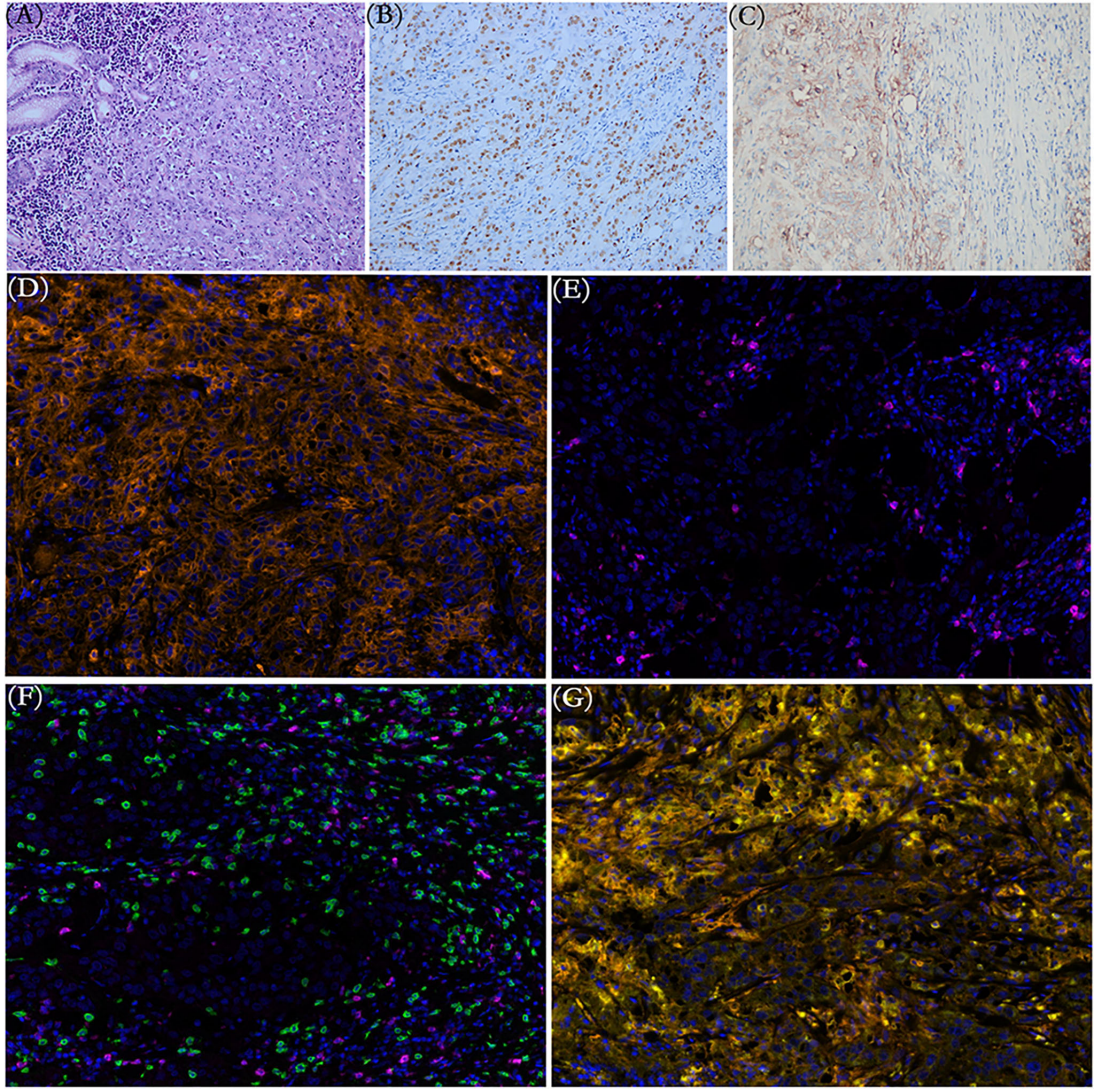
**(A)** Representative pathological image of gastric mass magnifications shows intense infiltrate of lymphocytes within the primary tumor (hematoxylin and eosin). **(B)** The brown cells in EBER-ISH ×200 magnification images are the cells harboring EBV infection (EBV-encoded small RNA *in situ* hybridization, EBER-ISH). **(C)** Immunohistochemical staining indicated broadly positive programmed death-ligand 1 (PD-L1) expression in the primary tumor. **(D–G)** Multiplex immunohistochemical analysis from a variety of cells in the tumor microenvironment. **(D)** PD-L1+ cells (orange). **(E)** PD-1+ cells (purple). **(F)** CD8+T cells (green). **(G)** PD-L1+ CD68+ macrophages (yellow). Original magnification ×200.

**Figure 2:**
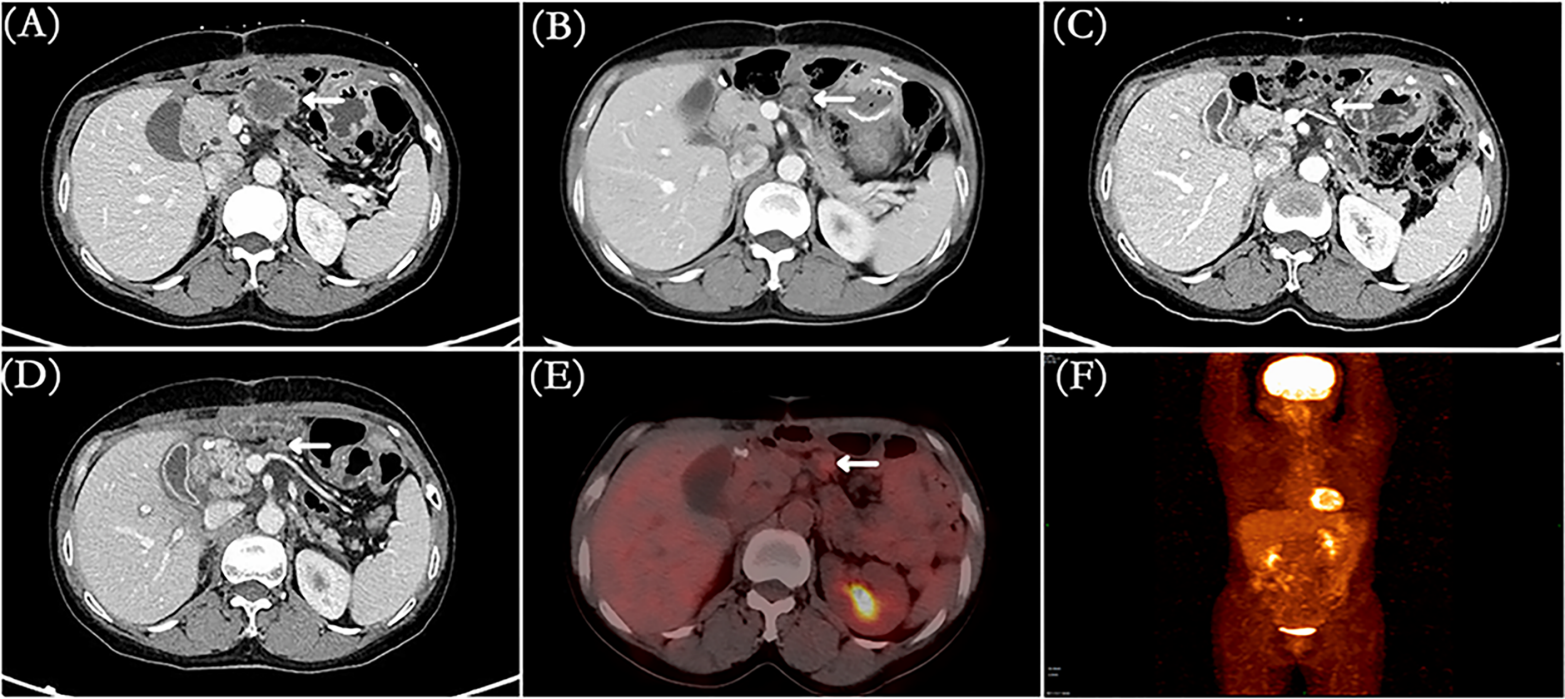
**(A)** CT image presented lymph node metastasis before treatment. **(B)** CT image showed early tumor shrinkage to PR after two cycles’ treatment. **(C)** CT image showed that lymph node metastasis was smaller (sustained PR) after four cycles. **(D–F)** CT and PET/CT images indicated lymph node metastasis with decrease >80% in size and mild uptake on 18F-FDG-PET/CT after seven cycles’ SOX + camrelizumab and three cycles’ S-1 + camrelizumab (total ten cycles). White arrows: lymph node metastasis.

A 56-year-old Chinese woman was admitted to our hospital emergency room in September 2020 with repeat fatigue, abdominal distension, and melena for 1 month. Abdominal computerized tomography (CT) suggested obviously uneven thickening and strengthening of the gastric body and gastric antrum wall, possibly accompanied by ulcers and multiple lymph nodes adjacent to the stomach enlarged. Gastroscopic examination revealed a giant ulcerative lesion located in the posterior wall of the gastric antrum, with the invasion of stomach angle and pylorus. Subsequent pathological examination of the biopsy showed poorly differentiated adenocarcinoma. On September 19, 2020, she underwent radical gastrectomy for distal gastric cancer and D2 lymphadenectomy. Pathohistological results of distal gastric cancer resection showed that the tumor was poorly differentiated adenocarcinoma with lymphoid stroma component (**Figure 1**), without any cancer in the surgical margin and metastasis to regional lymph nodes. An EBV-encoded RNA (EBER) assay demonstrated strong positive staining parallel to the tumor harboring EBV infection (**Figure 1**). Meanwhile, the immunohistochemistry indicated that MLH1, MSH2, MSH6, and PMS2 were overexpressed (pMMR) and human epidermal growth factor receptor 2 (HER2) was not amplified. The tumor was confirmed as pT4bN0M0 poorly gastric antrum differentiated adenocarcinoma with lymphoid stroma component (EBER-ISH+ and HER2-). The outcomes of next-generation sequencing (NGS) verified fifteen gene mutations (**Table 1**), a high tumor mutation burden (TMB) with 10.8 Muts/Mb, and microsatellite stable (MSS) status. Immunohistochemical (IHC) data of the tumor tissue suggested that the positive expression of PD-L1 protein and the tumor proportion score (TPS) was 70% and the combined positive score (CPS) was 75 (**Figure 1**). The immune microenvironment was examined by multiplex immunohistochemical staining and quantitative analysis (**Figure 1** and **Table 2**). Two months after the operation, abdominal CT showed enlargement of mass located in the gastrocolic ligament (**Figure 2**), which indicated metastatic lymph node (LN). Oxaliplatin 200 mg on day 1 plus oral S-1 60 mg twice a day, from days 1 to 14, along with camrelizumab 200 mg on day 1, repeated every 3 weeks, was administered as first-line treatment. Then, radiographic evaluation was performed every 8 weeks by enhanced CT. The significant resolution of the lymph node was observed after two cycles’ exposure of regimen SOX combined with camrelizumab, and the best efficacy evaluation was PR based on RECIST 1.1. Early tumor shrinkage was observed after 8 weeks, and persistent shrinkage of LN was achieved after 4 cycles (**Figure 2**). From then on, she had been exposed to SOX combined with camrelizumab up to 8 months and still achieved continuous PR. Moreover, the quality of life of the patient was good. Chemotherapy-associated AEs (grade 1 nausea, vomit and grade 2 anemia, grade 2 decreased neutrophil count, and decreased white blood cell count) were observed, and grade 1 reactive cutaneous capillary endothelial proliferation (RCCEP) was presented without any other immune-related adverse event. After 7 cycles’ SOX plus camrelizumab, the lesion was still unresectable due to whole abdominal adhesions. After multidisciplinary team (MDT) consultation, the patient underwent external-beam radiotherapy (EBRT) and received 50 Gy/25 fractions. She received S-1 and camrelizumab as the maintenance therapy up to 10 cycles followed by EBRT. Tumor is gradually and continuously shrinking in the latest visit and in deep response with a >80% decrease in size (**Figure 2**). Until now, PFS reached at least 12 months and the duration of response was beyond 10 months with manageable toxicity.

**Table 1:** The fifteen gene mutations of the patient.

Gene	Mutations	Mutation abundance (%)
PIK3CA	c.1634A>C p.E545A	10.93%
ABL1	c.3010C>T p.P1004S	8.51%
ARID1B	c.4296G>A p.M1432I	10.41%
CASP8	c.1414C>T p.R472*	10.86%
EPHA5	c.414C>A p.N138K	8.84%
FBXW7	c.1258C>T p.H420Y	8.79%
JAK2	c.1258C>T p.H420Y	8.79%
MLH3	c.1258C>T p.H420Y	11.7%
MYCL	c.1034C>T p.S345F	9.8%
NF2	c.1034C>T p.S345F	19.7%
PTPN11	c.1508G>T p.G503V	19.7%
RAD21	c.335A>C p.E112A	10.27%
RICTOR	c.872C>T p.T291I	2.16%
SOX9	c.872C>T p.T291I	0.46%
TET2	c.5059C>T p.Q1687*	0.69%

*means the termination codons. c.5059C>T p.Q1687* :aminoacids changes from Glutarnine(Q) to termination codons (*).

**Table 2:** Tumor-infiltrating immune cell test results.

Test indicators (multiplex IHC)	Test result
CD8+ T cells	+(10.59%)
PD-1+ cells	+(1.07%)
CD8+PD-1+ T cells	+(0.33%)
CD68+ macrophage cells	+(38.71%)
CD68+PD-L1+ macrophage	+(28.83%)

## Discussion

Searching for electrical databases, few clinical studies have focused on camrelizumab combined with chemotherapy as a first-line setting for gastric cancer, let alone for EBVaGC. As far as we know, this is the first report to show long-term response and safety of camrelizumab combined with chemotherapy in the first-line treatment of advanced EBVaGC. The cancer of the patient shown in this case quickly metastasized in a short period of time after the operation, which reflected the high degree of malignancy and poor biological behavior of the tumor. Then, early tumor shrinkage to PR was observed after two cycles’ exposure of camrelizumab combined with SOX regimen and persistency of response was observed. Notably, the median OS was 13.1 months in the nivolumab-plus-chemotherapy arm (patients with PD-L1 CPS ≥5) and the median PFS was 7.7 months (95% CI 7.0–9.2) with nivolumab plus chemotherapy in the CheckMate 649 study (2), in contrast, the PFS benefit (beyond 12 months) was more prominent in our case, which could be translated into a long-term survival benefit for this patient. The favorable responses of this patient may attribute to the unique characteristics of EBV-related cancers. Enriched tumor-infiltrating immune cells (lymphocyte and tumor-associated macrophages) exist in the EBVaGC microenvironment (9, 10). The density of CD68+macrophages was significantly higher in EBVaGC patients compared to Epstein–Barr virus-negative gastric cancer (EBVnGC), which was positively correlated with the expression rate of PD-L1 (11, 12). Compared with EBVnGC, the density of PD-L1+ tumor infiltrating immune cells was significantly greater in EBVaGC (10). Interestingly, several studies have shown that the quantity of PD-L1+CD68+macrophage may serve as an independent prognostic factor for survival or be significantly associated with favorable outcome to immunotherapy-based treatment in other malignancies, such as non-small-cell lung cancer, testicular lymphoma, and breast cancer (13–15). Hence, the quantity of PD-L1+CD68+macrophage may also serve as both an independent prognostic factor of EBVaGC and an effective predictor of EBVaGC in immunotherapy. Concordantly, through multiplex immunohistochemistry (mIHC), numerous infiltrated CD8+T lymphocytes and CD68+PD-L1+macrophages were observed in our case (**Figure 1** and **Table 2**), which provides a good antitumor environment. Next, the high levels of PD-L1 in cancer cells and inflammatory cells may be another interpretation for favorable outcomes (10). As the interaction of PD-L1 in cancer cells and programmed cell death protein 1 (PD-1) on the surface of T-cells enables tumor cells to escape from antitumor immunity, the high expression of PD-L1 in EBVaGC can be considered to be related to tumor progression (16). Accordingly, treatment with anti-PD-1/PD-L1 may prevent this interaction, thereby restoring the immune response against cancer cells. Thirdly, the high TMB in this patient may also play an important role in favorable outcomes. Although the predictive role of TMB in immunotherapy is still controversial, many immune-based studies indicated that due to its influence to invigoration of immune cells, patients with high TMB showed better curative effect to immune checkpoint inhibitors (ICIs) than those with non-high-TMB (6, 17).

Camrelizumab, a novel PD-1 inhibitor, possesses the characteristics of lower IC50 and EC50 values, increased affinity, and higher PD-1 receptor occupancy rate (>85%), which results in enhanced antitumor activity, compared to other PD-1 inhibitors (18, 19). Furthermore, camrelizumab showed impressive efficacy and manageable toxicity in a wide spectrum of solid tumors, including Hodgkin lymphoma (20), B-cell lymphoma (21), esophageal squamous cell carcinoma (22), gastric and gastroesophageal junction cancer (23), hepatocellular carcinoma (23), nasopharyngeal cancer, and non-squamous, non-small cell lung cancer (24). Hence, the possibility of long-term tolerance coming from low toxicity should be taken into account. No serious adverse events have been shown during the treatment outside of grade 1 nausea and vomit, grade 2 anemia, decreased neutrophil count, and decreased white blood cell count, which were more prone to chemotherapy-related toxicities. Under prolonged exposure to camrelizumab, mild RCCEPs were observed without any other immune-related adverse event. Further follow-up is needed.

The complexity of the relationship between cancer and the immune system renders it difficult to identify a single predictive biomarker. Although favorable clinical outcomes were observed in patients with EBV-negative PD-L1 positive treated with chemotherapy plus PD-L1 antibody in a published study (25), PD-L1 expression levels might not be a robust predictor for anti-PD-1/PD-L1 therapies in GC (25–27). Commonly, EBVaGC patients showed a favorable clinical outcome to immunotherapy, and almost EBV+GC cases presented high PD-L1 CPS (28, 29), just as this patient with CPS = 75, and this phenomenon was seldom seen in EBVnGC. Hence, we believed that the EBV-positive status might be a superior predictor than PD-L1 for immunotherapy in GC (7, 30). Could the combination of different factors become a more accurate biomarker? High PD-L1, enriched PD-L1+CD68+macrophages, and high TMB were presented in this patient with EBVaGC. Combination of multiple biomarkers could have a higher efficacy predictive capacity to immunotherapy. This speculation was consistent with a previous study in which 3 patients with EBVaGC showing PR were PD-L1 positive and at the last follow-up, their durations of the response were 13.8, 18, and 10 months, respectively (7), whose finding highlighted the long-lasting nature of immunotherapy. This assumption requires further large-scale clinical trials for verification.

To our knowledge, this is the first case with high PD-L1 (TPS = 70%, CPS = 75), enriched PD-L1+CD68+macrophages (28.83%), and high TMB (10.8 Muts/Mb). EBVaGC was treated with camrelizumab combined with oxaliplatin and S-1 as the first-line therapy. Early tumor shrinkage, deep response, durable PFS, and manageable toxicities were exhibited. Moreover, multidisciplinary approaches—camrelizumab plus SOX (induction therapy), radiotherapy (local therapy), and camrelizumab plus S-1 (maintenance therapy)—might be the more suitable integrated treatment for this patient with oligometastatic lesion. It remained unclear, however, whether prominent PD-L1+CD68+macrophages were a common finding in EBVaGC or just for our patient. Next, cross talk among PD-L1+CD68+macrophages, T cells, and cancer cells was unknown. Additionally, repeated mIHC examinations, although better, were difficult to apply in real-world clinical practice.

In conclusion, the present study suggests that camrelizumab combined with SOX might be a promising and well-tolerated regimen as the first-line treatment in metastatic EBVaGC with high PD-L1 CPS and enriched PD-L1+CD68+macrophages in the tumor microenvironment. For a highly heterogeneous malignancy, we recommend gene sequencing and multiplex immunohistochemical to find a new strategy. It deserves prospective research to further validate the efficacy.

## Data Availability Statement

The original contributions presented in the study are included in the article/supplementary material. Further inquiries can be directed to the corresponding author.

## Ethics Statement

The studies involving human participants were reviewed and approved by West China Hospital of Sichuan University Biomedical Research Ethics Committee. The patients/participants provided their written informed consent to participate in this study. Written informed consent was obtained from the individual(s) for the publication of any potentially identifiable images or data included in this article.

## Author Contributions

XL and HL performed the radiological analysis of CT images. LF, CC, and JL collected the clinical data. WL and KC wrote the first draft of the manuscript. DC wrote the sections of the manuscript. All authors contributed to the article and approved the submitted version.

## Funding

This research was partly supported by Sichuan Province Health Planning Committee Research Project (No. 19PJ083).

## Conflict of Interest

The authors declare that the research was conducted in the absence of any commercial or financial relationships that could be construed as a potential conflict of interest.

## Publisher’s Note

All claims expressed in this article are solely those of the authors and do not necessarily represent those of their affiliated organizations, or those of the publisher, the editors and the reviewers. Any product that may be evaluated in this article, or claim that may be made by its manufacturer, is not guaranteed or endorsed by the publisher.
